# Systemic sclerosis (positive anti-Ro 52 and anti-centromere antibodies) in a patient after COVID-19 infection: a rare case report

**DOI:** 10.1097/MS9.0000000000002032

**Published:** 2024-04-15

**Authors:** Paras Oli, Prabhat Poudel, Shradha KC, Aastha KC, Anil Kumar Sah, Pankaj Yadav

**Affiliations:** a Department of Internal Medicine(Neurology), Nepal Medical College; b Department of Internal Medicine, Kathmandu Medical College; c Department of Internal Medicine, Institute Of Medicine, Kathmandu; d Department of Internal Medicine, Chitwan Medical College, Chitwan; e Department of Internal Medicine, BP Koirala Institute Of Health Sciences, Dharan, Nepal

**Keywords:** autoimmune disease, post-COVID-19, systemic sclerosis

## Abstract

**Introduction and importance:**

The SARS-CoV-2 is the source of COVID-19, a respiratory disease. It typically manifests as restricted pulmonary symptoms, but autoimmune dysfunction might occasionally show up. A COVID-19 infection may cause a multi-system connective tissue disease known as systemic sclerosis (SSc). In patients who recovered from COVID-19, autoimmunity may have multiple underlying causes.

**Case presentation:**

The authors report the case of a 68-year-old female who, 1 month after contracting COVID-19, complained of dyspnoea and muscle exhaustion. The patient was treated for post-COVID syndrome. She developed symptoms of chronic dyspnoea, pale fingers, pursed lips, trouble chewing and swallowing, and muscle weakness after 7 weeks. A chest high-resolution computerised tomography (HRCT) scan suggested interstitial lung disease. Clinical characteristics and an autoantibody profile containing anti-Ro 52 and anti-centromere antibodies pointed towards SSc. She was treated with azathioprine and prednisolone at a reduced dosage, and she is now stable with monthly follow-ups.

**Clinical discussion:**

COVID-19 might induce cytokine storms and immunological dysregulation, ultimately culminating in autoimmune manifestations. Several autoantibodies are observed in autoimmune illnesses in post-COVID-19 infection patients. Our situation is distinct because SSc following a COVID-19 infection is not commonly seen as an autoimmune illness.

**Conclusion:**

The number of patients with rare autoimmune diseases, like SSc, following COVID-19 has been rising. Therefore, we should consider the possibility of autoimmune disease when looking into a patient who presents strangely or has developed new symptoms after COVID and should contact the patient’s management immediately.

## Introduction and importance

Highlights

- COVID-19 infection can lead to immune dysfunction leading to various autoimmune diseases.
- Systemic sclerosis manifestation after COVID-19 infection is a rare complication of post-COVID-19 immune dysfunction.
- Patient presenting with unique symptoms after COVID-19 infection must be dealt keeping autoimmune disease in mind for early diagnosis and management.

Systemic sclerosis (SSc) is an autoimmune connective tissue disease affecting multiple systems, mainly characterised by hallmarks of autoantibody production, vasculopathy of small vessels, and abnormal fibroblast proliferation resulting in increased extracellular matrix deposition^1^. It is a rare but life-threatening disease with no definitive cure^2^. It is more common in young and middle-aged females^3^. A recent systematic review and meta-analysis showed a prevalence of SSc to be 17.6 per 100 000 and an incidence rate of 1.4 per 100 000 person-years^4^. The pathogenesis of SSc is unknown and is believed to be multifactorial, including genetic, environmental, and altered immune regulation. Occupational exposure to silica and hydrocarbons has also been linked in some studies^5^. As a part of innate immunity, type 1 interferon (IFN) has a role in tackling viral and bacterial infections. Research has pointed to the role of type 1 IFN and IFN-inducible genes in the pathogenesis of SSc^6^. Viral infection has been shown to disrupt the immune system, resulting in autoimmunity, mainly through molecular mimicry and IFN activation^7^. Here, we present the case of a 68-year-old female who, after the COVID-19 infection, had immune dysregulation, resulting in an autoimmune disorder called SSc.

## Case presentation

A 68-year-old woman arrived at our emergency room complaining of dyspnoea, abdomen pain, and muscle weariness that had persisted for a month. Only during physically demanding tasks, such as climbing stairs, did dyspnoea occur. Two months prior, she had a history of COVID-19 infection with the saturation of 90%, and she was very anxious because of the COVID-19 infection at the time of her admission, which returned to baseline within a few hours after proper counselling and oxygen therapy. She spent a week in the hospital; her stay was uneventful. She has a history of four years of diabetes mellitus (DM), 10 years of hypertension (HTN), and occasional episodes of gastritis, for which she is taking omeprazole 20 mg as needed. For her DM and HTN, she takes 500 mg of metformin once daily and 5 mg of amlodipine once daily, respectively. There was no history of cardiorespiratory disease in her family. Her blood pressure was higher (140/90 mmHg) during the assessment. Vital signs, including O_2_ saturation, were normal. Examining the chest, typical vesicular sounds were heard.

The results of the lab tests were normal, and the chest X-ray revealed minor opacification around each lung’s periphery, which was assumed to be a result of COVID-19 alterations. Proton pump inhibitors were used to treat the patient’s abdominal pain, the post-COVID syndrome was identified, and the patient received status counselling. A month later, the patient had the same symptoms: ongoing muscle weakness, chest pain, exertional dyspnoea, a productive cough for a week, and 2 days of fever. Her temperature was 101.8 °F, and her saturation was 92% upon physical examination. During the physical examination, the right lower lobe’s bronchial breathing and the bilateral lung fields both showed coarse crackles. A chest X-ray revealed consolidation on the lower lobe of the right lung. She was diagnosed with community-acquired pneumonia and was treated with antibiotics and oxygen therapy. After clinical and radiological improvement, she was discharged after 8 days.

**Figure 1:**
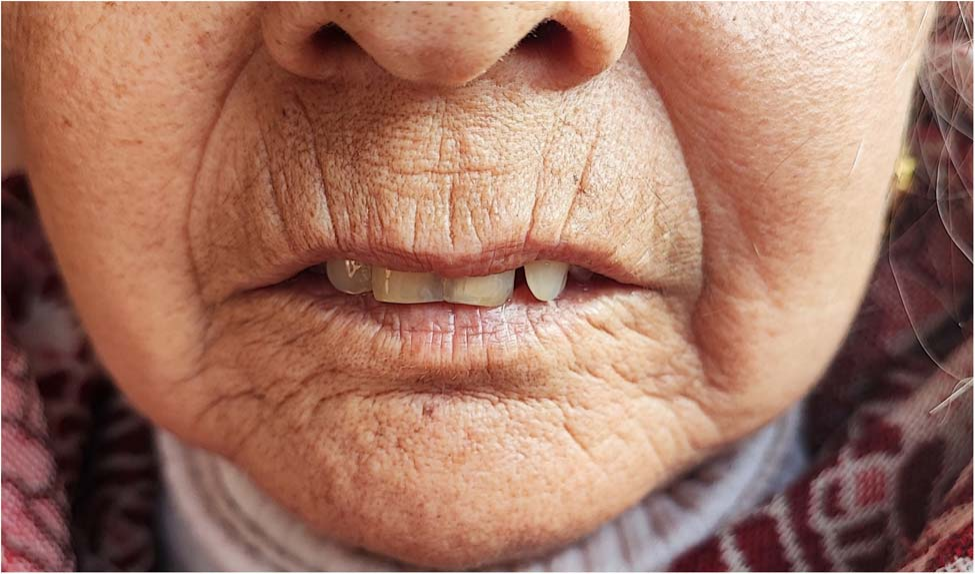
Scleroderma facies with pursed and tightened lips with increased visibility of teeth.

**Figure 2:**
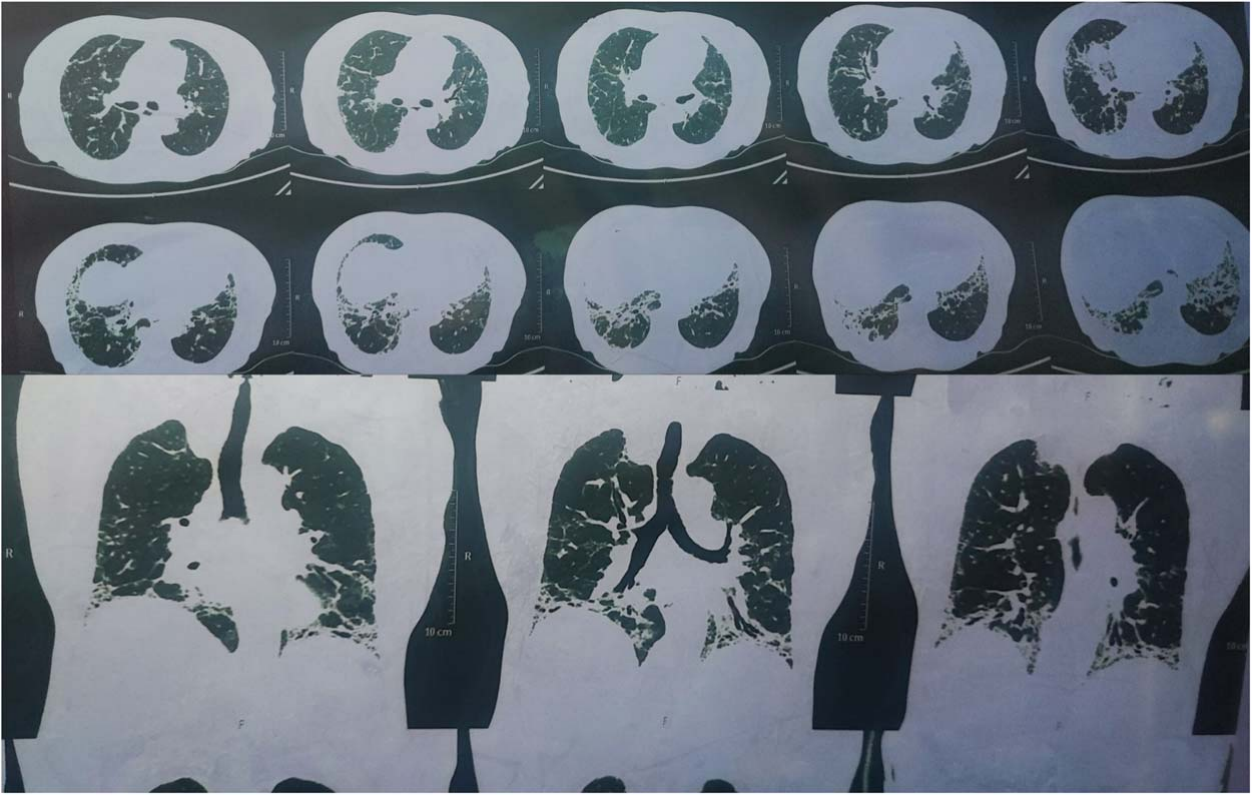
High-resolution computerised tomography scan showing a honeycombing pattern with fibrotic and traction bronchiectatic changes on bilateral lungs.

The patient complained of dyspnoea, ongoing muscle fatigue, multiple joint pain, difficulty swallowing, pursed and tightened lips with increased visibility of teeth (Figure 1), burning sensations in all four limbs, and pale fingers when exposed to cold after 2 weeks. The patient eventually made an appointment with our general outpatient department. We were reminded of some autoimmune conditions when we were discussing post-COVID syndrome during this presentation. The results of the lab study indicated a positive rheumatoid factor and an erythrocyte sedimentation rate (ESR) of 90 mm/h. The level of vitamin B-12 was low, may be because of her vegetarian diet, and she also had a recurrent history of low vitamin B-12 in the past. An X-ray of the chest showed bilateral lungs with fibrotic alterations and reticular opacity, along with an elevated left hemidiaphragm. Due to persistent dyspnoea, the patient was scheduled for a spirometry and high-resolution computed tomography (HRCT) scan of the chest. Spirometry revealed a restrictive pattern of lung disease, and the HCT scan showed a honeycombing pattern with fibrotic and traction bronchiectatic changes on bilateral lungs suggestive of interstitial lung disease (ILD) (Figure 2). Anti-CCP and uric acid levels were within the normal range. The autoantibody profile showed positive Ro-52 recombinant and centromere B (CB) antibodies, whereas Scl-70 was negative (Table 1).

**Table 1:** Antibody profile.

Antigen	Intensity	Class (0, (+), +, ++, +++)
RNP/Sm (RNP/Sm)	1	0
Sm(Sm)	1	0
RNP 70 (70)	1	0
RNP A (A)	1	0
RNP C (C)	1	0
SS-A native(60 kDa) (SSA)	0	0
Ro-52 recombinant (Ro-52)	113	+++
SS-B (SSB)	1	0
Scl-70 (Scl)	1	0
PM- Scl 100 (PM 100)	2	0
Jo-1 (Jo)	0	0
Centromere B (CB)	128	+++
PCNA (PCNA)	1	0
dsDNA (dsDNA)	0	0
Nucleosomes (NUC)	1	0
Histones (HI)	1	0
Ribiosomal Protein (RIB)	3	0
AMA-M2 (M2)	2	0
Control (Ko)	108	+++
Label (ET)	-1	
Intensity	Class	Explanation
0–5	0	Negative
6–10	(+)	Borderline
11–25	+	Positive
26–50	++	Positive
51–256	+++	Strong positive

The diagnosis of SSc was made following “The American College of Rheumatology/European League Against Rheumatism criteria for the classification of systemic sclerosis (SSc)”. The patient was referred for an ophthalmologic consultation and a normal Schirmer test. She was started on a decreasing dose of prednisolone, starting at 30 mg for 2 weeks, and then continued on 10mg. Vitamin B-12 and calcium supplements were also started, and the Raynaud phenomenon was managed with nifedipine. Follow-up after a month showed her symptoms of muscle weakness and dysphagia reduced significantly, and her vitamin B-12 level returned to a normal level after supplementation. The patient and her family were counselled regarding exposure to cold weather and the status and nature of her disease. Her blood sugar level was slightly high. Her serum is still positive for autoantibody, but with clinical improvement. She had increased dyspnoea with an oxygen saturation of 85% after 1 month of her initial follow-up, for which she was admitted for 2 more days and was started on azathioprine 50mg daily at discharge. She is currently stable and is on regular monthly follow-up.

## Clinical discussion

COVID-19 is a respiratory illness that caused a pandemic in 2019 caused by the SARS-CoV-2 virus. Its disease spectrum can range from asymptomatic cases to severe pneumonia. Because of COVID-19 characteristics and immune activation, it has been linked with many autoimmune and rheumatological manifestations^8^. Microbial infection leading to autoimmune manifestation is backed up by three mechanisms: molecular mimicry, epitope spreading, and bystander activation^9^. Many autoantibodies are seen in autoimmune diseases due to the COVID-19 infection, which is more prevalent in the older population^10^. Antineutrophilic antibody (ANA) was seen in 4–50% of COVID-19 infection patients^8^. The COVID-19 infection results in cytokine storms and immune dysfunction. Cytokines like interferon (IFN), interleukin (IL)-6, IL-8, IL-1, etc. lead to multi-organ dysfunction and autoimmunity^8^. Many autoimmune diseases that manifest in post-COVID-19 patients are systemic lupus erythematosus (SLE), polyarteritis nodosa, inflammatory myopathy, Graves disease, rheumatoid arthritis, immune thrombocytopenic purpura, vasculitis, and Guillain-Barre syndrome (GBS)^11^. Accurate data on new-onset SSc post-COVID-19 is unavailable, making our case a rare scenario.

SSc, or scleroderma, is an autoimmune disease leading to abnormal proliferation of fibrous tissue and deposition of extracellular matrix in different body parts. Extracellular matrix deposition due to fibroblast dysfunction, hypoxia due to vascular abnormality, and lymphocyte dysfunction leading to autoantibody production are the hallmarks of SSc^1^. Scleroderma can be divided into limited cutaneous systemic sclerosis (lcSSc) and diffuse cutaneous systemic sclerosis (dcSSc) based on the distribution of skin thickening. Vascular manifestation is the earliest and almost universal manifestation of SSc^12^. Another clinical manifestation depends on the systems involved, like puffy oedematous skin, cardiovascular conduction abnormality, pulmonary hypertension in cardiorespiratory involvement, dysphagia, gastrointestinal reflux disease, bleeding disorders like gastric antral vascular ectasia (GAVE) in cases of gastrointestinal involvement, scleroderma renal crisis presenting as hypertensive emergencies (e.g. acute pulmonary oedema and microangiopathic haemolytic anaemia), new-onset renal failure in cases of renal involvement, myalgia, arthralgia, arthritis, contracture, and carpal tunnel in cases of musculoskeletal involvement, and many more impacting quality of life^13^. Autoantibodies against antigens like topoisomerase I, centromere protein (CENPs), PM/Scl complex, or RNA polymerase III can be seen in up to 95% of patients^14^. There are two main types of SS-A/Ro-associated antibodies: SS-A/Ro60 and Ro-52/TRIM 21^14^. Our patient has positive Centromere protein B (CENP-B) and Ro-52 autoantibodies. As per the Canadian Scleroderma Research Group (CSRG) cohort of SSc patients, 36% and 20% were CENP-B and Ro-52 positive, respectively^14^. In patients with new-onset SSc, antibodies targeting Ro-52 are found abundantly in the lungs, resulting in loss of lung function over time, which increases linearly with an increase in antibody levels^15^. SSc-ILD (interstitial lung disease) is one of the major complications leading to morbidity and mortality, with variable progression rates^15^.

The latest guideline of the American College of Rheumatology/European League Against Rheumatism (ACR/EULAR) criteria for the classification of SSc helped us in making the diagnosis of SSc in our patient with a score of 10 (Raynaud’s Phenomenon:3, SSc-related autoantibodies:3, ILD:2, Puffy Fingers:2). COVID-19 usually manifests in phases, starting with the asymptomatic phase in the early weeks and progressing to the inflammatory and hypercoagulable phases, leading to organ dysfunction^3^. Earlier asymptomatic patients before COVID-19 infection and the timing of symptoms post-COVID in our patient overlapped with the inflammatory/autoimmunity period of COVID-19, which helps us in diagnosing SSc, most likely as a complication of COVID-19. The mechanism of autoimmunity in COVID-19-infected patients is still debatable. Still, cases like ours are on the rise post-COVID, which requires a detailed immunological and genetic study to tackle autoimmune manifestations in COVID-19-infected patients in the future.

## Conclusion

Autoimmune manifestations are on the rise among patients after the COVID-19 infection. Autoimmune infections like SSc post-COVID-19 in themselves are rare. Diagnosing autoimmune disorders post-COVID is difficult due to the overlapping manifestations of different phases of the COVID-19 infection. Like in our case, many cases are tackled by keeping post-COVID syndrome as a diagnosis, which can create challenges while managing cases after an accurate diagnosis. So, patients presenting with unique and unclear symptoms after COVID-19 infection must be tackled, keeping autoimmune manifestations in mind, as they may hide in long-term COVID-infected patients. Early diagnosis by analysis of autoantibodies and proper management are essential to prevent SSc-related morbidity and mortality.

## Ethical approval

None.

## Consent

Written informed consent was obtained from the patient for publication of this case report and accompanying images. A copy of the written consent is available for review by the Editor-in-Chief of this journal on request.

## Source of funding

None.

## Author contribution

P.O.: literature review, manuscript preparation, review. S.K.C.: manuscript preparation, review. A.K.C.: manuscript preparation, review. P.P.: manuscript preparation, review. A.K.S.: manuscript preparation, review. P.Y.: manuscript preparation, review.

## Conflicts of interest disclosure

The author declares no conflicts of interest.

## Research registration unique identifying number (UIN)

None.

## Guarantor

Paras Oli.

## Provenance and peer review

Not commissioned, externally peer-reviewed.
